# A review of modern insulin analogue pharmacokinetic and pharmacodynamic profiles in type 2 diabetes: improvements and limitations

**DOI:** 10.1111/j.1463-1326.2011.01395.x

**Published:** 2011-08

**Authors:** M Evans, P M Schumm-Draeger, J Vora, A B King

**Affiliations:** 1 Department of Medicine, University Hospital of WalesCardiff, UK; 2 Clinic for Endocrinology, Diabetes and Angiology, Bogenhausen Academic Teaching HospitalMunich, Germany; 3 Department of Diabetes and Endocrinology, Royal Liverpool University HospitalLiverpool, UK; 4 Diabetes Care CenterSalinas, CA, USA

**Keywords:** insulin analogues, insulin therapy, pharmacology, type 2 diabetes

## Abstract

Insulin analogues have been engineered to enhance desired molecular properties without altering immunogenicity. The majority of insulin pharmacology studies are conducted in healthy volunteers and patients with type 1 diabetes. At present, there are more patients with type 2 than type 1 diabetes receiving insulin treatment. As the responsibility for initiating insulin therapy in these patients continues to shift to primary care, it will be important for general practitioners to understand the different pharmacological properties of insulin preparations in patients with type 2 diabetes, so that treatment can be adapted to meet patients’ physiological and lifestyle requirements. The purpose of this review is to summarize pharmacological studies of insulin analogues in patients with type 2 diabetes. Faster onset of action of rapid acting insulin analogues has improved postprandial glycaemic control. Biphasic insulin analogues are associated with a lower incidence of nocturnal hypoglycaemia compared with human biphasic preparations and allow for intensification from once to twice or thrice daily dosing. More predictable glycaemic-lowering profiles of the insulin analogues have also led to reductions in nocturnal hypoglycaemia, particularly comparing long-acting insulin analogues with protaminated human insulin. Enhancing insulin self-association and reversible binding with albumin has led to further reductions in variability. However, improvements can still be made. Effective once daily clinical dosing of long-acting insulin analogues is not possible in all patients. In addition, the protaminated component of biphasic insulin analogues do not provide the duration of action or profile for physiological basal insulin replacement and neither insulin glargine nor insulin detemir are suitable for mixing with other insulin analogues as this would substantially alter their pharmacokinetic properties. Enhancing the pharmacological predictability and extending the duration of action could simplify insulin titration and further reduce the incidence of hypoglycaemia.

## Introduction

Insulin, a 51 amino acid 5.7 kDa protein comprised of an A and B chain linked by two di-sulphide bridges and produced by pancreatic islet β cells, is one of the best-known and most-studied proteins. First isolated in 1921 and used as a treatment of type 1 diabetes in 1922, insulin has advanced from early animal to biosynthetic human and analogue preparations and is increasingly used to treat type 2 diabetes at various stages of disease progression. The advance from animal to human preparations had obvious advantages linked to sequence homology with the native human protein, reducing the incidence of antibody formation that led to injection site reactions and blocking of therapeutic effect. More recently, insulin analogues have been engineered to enhance desired molecular properties (e.g. rapid absorption or prolonged duration of action) without altering immunogenicity. These improvements have enabled physicians to tailor treatment regimens and more closely emulate the normal insulin physiology comprised of a stable basal secretion with surges of insulin closely temporally related to food ingestion (reaching maximal concentrations 45–60 min and returning to baseline within 2–3 h) [1].

Studies of pharmacokinetics and pharmacodynamics are an integral part of clinical drug development and are particularly important to insulin clinical development where the aim has been to mimic the normal physiology of insulin secretion and action in relation to and between meals. Most of these studies are conducted in healthy volunteers (low doses) to ascertain safety and patients with type 1 diabetes (treatment doses) to describe more fully, insulin metabolism and glucose lowering effect. As patients with type 1 diabetes have no/negligible endogenous insulin, the measurements of insulin, glucose and other metabolic effects are ascribed to the administration of exogenous insulin.

The site of injection [2], thickness of the subcutaneous tissue [3,4], subcutaneous blood flow [5–7] and the amount of insulin administered [8] have all been reported to influence human insulin pharmacokinetics. In addition, human insulin pharmacokinetics has been shown to be different in patients with type 2 compared with type 1 diabetes and may also be altered by the presence of obesity. Clauson and Linde [9] reported 15–45% slower absorption kinetics of human insulin (Actrapid) in obese and normal body weight type 2 diabetes patients relative to previously studied patients with type 1 diabetes. Just as in patients with type 1 diabetes, the highest rates of absorption were seen following subcutaneous administration to the epigastric area in normal body weight patients with type 2 diabetes, however, no differences in absorption rates between various injection sites were seen in the obese patients with type 2 diabetes. Ryysy et al. [10] found that increases in total body adiposity rather than subcutaneous thickness were most closely associated with insulin dose and delayed insulin absorption.

With the number of patients with type 2 diabetes receiving insulin treatment presently exceeding the number of patients with type 1 diabetes and the responsibility for initiating insulin therapy in these patients continuing to shift to primary care [11], it will be increasingly important for general practitioners to understand the pharmacological properties of currently available and emerging insulin preparations in type 2 diabetes patients, so that treatment can be adapted to meet patients’ physiological and lifestyle requirements.

This review summarizes pharmacokinetic and pharmacodynamic studies of insulin analogues performed in patients with type 2 diabetes.

## Methodology

Relevant publications were identified by means of a PubMed literature search using the following criteria: (‘insulin’) AND (‘lispro’ OR ‘aspart’ OR ‘glulisine’ OR ‘glargine’ OR ‘detemir’) AND (‘type 2 diabetes’) AND (‘pharmacokinetic’ OR ‘pharmacodynamic’). Other studies were identified from the bibliography of short-listed articles. The search was limited to English language publications. An initial review of all titles and abstracts was performed for relevance. If deemed appropriate for further review, access to the full article was obtained. The figures included in this review relating to insulin concentration, glucose infusion rate (GIR) and blood glucose profiles were reconstructed from previously published figures using the semi-automated software, Digitize It v1.5 (Eden Prairie, Minnesota, USA) [12].

## Insulin Analogues

Under normal physiological circumstances, insulin is synthesized in the pancreatic β cells and stored in vesicles of the Golgi-apparatus in relatively high quantity. In the vesicles of the β cell insulin is stored as hexamers, which form as a result of the high local insulin concentration and the presence of zinc. When released to the circulation, these insulin hexamers dissociate to form dimers and monomers, because of the dilution effect and zinc diffusing away. Insulin molecules in solution exist in dynamic equilibrium as monomers, dimers, double dimers (tetramers), triple dimers (hexamers) and higher-order aggregates. The extent of insulin self-association is dependent on factors that include insulin concentration, zinc concentration and pH. Altering the solvent composition and the primary structure of insulin can also affect the charge and solubility of the insulin molecule.

Before the advent of insulin analogues, the principal method of delaying absorption of injectable insulin preparations was through adding protamine and/or zinc to stabilize insulin hexamers [13]. The breakdown of protamine and/or dissipation of zinc following subcutaneous injection de-stabilizes the hexamers and results in the slow release of dimers and monomers. The addition of protamine and zinc is not a process limited to animal and human insulin, but is also seen in production of some insulin analogue preparations. Biphasic insulin analogues, for example, comprise a mixture of soluble and protaminated or precipitated insulin lispro or insulin aspart in a range of specific ratios.

As insulin monomers and dimers enter the capillary system at a faster rate than hexamers and only monomeric or dimeric insulin is biologically active at the insulin receptor, the speed of hexamer dissociation determines, to a large extent, the observed pharmacokinetic (circulating insulin concentration) and pharmacodynamics (glucose lowering) profiles of an insulin preparation [14,15]. Soluble human insulin also consists of hexamers, which dissociate into dimers and monomers at the injection site. The lag phase between injection of hexamers and the availability of biologically active dimers and monomers at target tissues, necessitates that soluble human insulin be given 30 min or so before meal consumption [16]. In addition, identical doses of insulin do not result in identical pharmacological response either between subjects or within the same individual at different times. This between- and within-subject variability in pharmacological action, which in turn results in unpredictable effects with hypoglycaemia and hyperglycaemia, is particularly problematic with human insulin preparations. These limitations have fuelled the development of insulin analogues.

**Figure 1:**
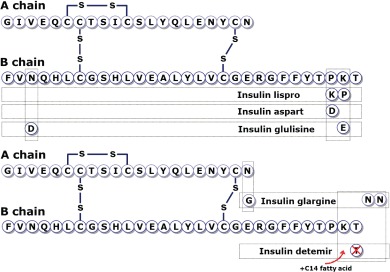
Summary of the changes made to the insulin molecule using single letter coding for amino-acids. A, alanine; C, cysteine; D, aspartic acid; E, glutamic acid; F, phenylalanine; G, glycine; H, histidine; I, isoleucine; K, lysine; L, leucine; M, methionine; N, asparagine; P, proline; Q, glutamine; R, arginine; S, serine; T, threonine; V, valine; W, tryptophan; Y, tyrosine.

Broadly speaking there are currently two different categories of insulin analogues: rapid acting insulin analogues (insulin lispro, insulin aspart and insulin glulisine); and long-acting insulin analogues (insulin glargine and insulin detemir). Biphasic insulin analogues are a mixture of soluble and protaminated or precipitated forms of either insulin lispro or insulin aspart. Changes to the insulin molecule have focused on the B chain, away from receptor binding elements. With the exception of insulin detemir, the other insulin analogues have been developed through the substitution or addition of amino acids that result in changes to the charge and/or conformation of the insulin molecule at physiological pH (figure 1). Insulin lispro is formed by switching lysine and proline amino acids at positions B28 and B29 and insulin aspart by substituting proline with a negatively charged aspartic acid in position B28. Insulin glulisine differs from human insulin by substituting aspargine for lysine at position B3 and lysine for glutamic acid at position B29 and unlike the other two rapid-acting analogues, does not contain zinc. Rapid-acting insulin analogue modifications weaken the propensity for the insulin to self-associate through charge repulsion at the sites where insulin monomers would normally associate to form dimers, resulting in rapid absorption of monomers from the subcutaneous tissue at the time of injection [17]. Insulin glargine is formed by replacing asparagine with glycine at position A21 and lengthening the B chain by adding two arginine amino acids at the C terminus. These modifications shift the isoelectric point (the pH at which the molecule carries no electrical charge) towards a more neutral pH, making it less soluble at physiological pH and promoting precipitation following subcutaneous injection. Subsequent enzymatic removal of the two arginine amino acids at the B chain terminus occurs at a variable rate, both at the site of injection and in the circulation, yielding products which remain metabolically active at the insulin receptor [18].

Insulin detemir is unique in the sense that a C14 fatty acid has been covalently bonded to lysine in position B29 and the terminal threonine of human insulin in position B30 has been removed. This fatty acid side chain not only increases self-association into hexamers and di-hexamers, but also allows reversible binding of insulin detemir to albumin present in all tissue and in the circulation. Albumin binding contributes almost as much to the prolongation of action of insulin detemir, as does the di-hexameric self-association [19]. This approach to prolonging duration of action also confers several other advantages such as a buffering of insulin concentration resulting in improved predictability, as well as hypothesized hepato-selectivity which might explain the weight sparing effects reported with this insulin [20].

## Mitogenicity

There is continuing debate concerning the risk of cancer and the association with insulin use in patients with type 2 diabetes. Both the presence of obesity and diabetes are documented, risk factors for the development of cancer per se. Insulin may also potentially increase the risk of cancer through growth promoting and anabolic effects mediated by binding to the type 1 insulin-like growth factor (IGF) receptor [21]. The type 1 IGF receptor binding has been linked to tumour development in rodents [22] and these findings have led to the discontinuation of specific insulin analogues. Currently available insulin analogues exhibit an affinity for the type 1 IGF receptor ranging between 16 and 641% relative to native human insulin, depending on the specific insulin analogue and cell line studied [23,24,57]. The speed of insulin dissociation from the insulin receptor may also contribute to the mitogenic potential of insulin analogues[23].

A series of recently published epidemiological studies have attempted to investigate the effects of insulin use on cancer risk [25–31]. These studies have reported mixed results. The largest study of 127 031 patients reported an increase in the risk of cancer with higher doses and longer duration of exposure to any insulin and that after adjusting for insulin dose, insulin glargine-treated patients were at highest risk [26]. Another study of 114 842 patients reported an association between insulin glargine used alone and increased risk of breast cancer [27]. Currie et al. [31] also reported an association between the use of any insulin and cancer risk relative to patients receiving oral antidiabetic drug treatment, with no additional attributable risk of insulin glargine over other (predominantly human) insulin preparations. Lastly, a smaller study of 12 842 patients followed for 4 years also found an association between exclusive insulin glargine use and the risk of cancer, but not overall use of insulin glargine [28]. These epidemiological studies, however, are liable to a number of inherent weaknesses in study design, the main limitation being the inability to control for confounding factors which importantly included weight, duration of diabetes and smoking in some instances. No evidence for increased cancer risk has been reported during an extended trial investigating the effects of neutral protamine Hagedorn (NPH) insulin and insulin glargine effects on the development of retinopathy [29] or in the meta-analysis of 31 insulin glargine studies [30]. Similarly, a meta-analysis of trials comparing insulin detemir and insulin glargine reported comparable risk of malignancy between each other and to NPH [25]. However, as these *post hoc* analyses are based on limited duration trials that were not specifically designed to investigate mitogenic potential, these findings should also be interpreted with caution.

The recently published consensus guidelines issued by the American Diabetes Association and American Cancer Society conclude that the evidence for specific diabetes drugs affecting the risk of cancer is limited and that overriding diabetes indications rather than putative cancer concerns should remain the principal consideration when selecting therapy in patients with type 2 diabetes [32].

## Pharmacokinetics

Several pharmacokinetic parameters describe the absorption, distribution, metabolism and elimination of a drug. Insulin absorption is derived principally through the measure of circulating insulin concentration in relation to time since subcutaneous injection. The evaluation of insulin pharmacokinetics has traditionally been undertaken by the measurements of plasma immunoreactive insulin levels after administration of the preparation in question. Such studies are conducted in normal subjects and patients with type 1 diabetes. Pharmacokinetic action profiles have also been evaluated using the euglycaemic clamp technique in normal subjects and patients with type 1 and 2 diabetes, whereby GIRs are examined during maintenance of plasma glucose levels at a predetermined value. These systemic measurements have also been strengthened by measurements of disappearance of radioactivity after subcutaneous administration of various radiolabelled insulin preparations. Together, the measures provide a complete description of the way in which the body processes the insulin from administration to elimination.

Ideally, insulin concentrations should show dose proportionality (i.e. for an increase or decrease in the administered dose, there is a proportional increase or decrease in the insulin concentration) and the profile of insulin concentration over time should resemble the desired glucose lowering profile. Thus for rapid-acting insulin analogues, the insulin concentration should rise to a peak very quickly after injection and be rapidly metabolized to inactive metabolites and eliminated. Duration of action of currently available rapid acting insulin can vary between 3 and 5 h depending on the dose injected [33]. For long-acting insulin analogues, the insulin concentration should be consistent and stable. It should be noted, however, that the waning insulin concentration of current basal insulin analogues may not provide for the increase in morning insulin needed to cover the transient morning increase in insulin resistance (i.e. the dawn phenomenon) [34]. For biphasic insulin analogues, the ideal profile would be a combination of both a rapid onset and peak insulin action following injection to cover the postprandial period, followed by a rapid return to a long flat profile to cover basal needs. Current biphasic formulations are not ideal in this respect.

**Figure 2:**
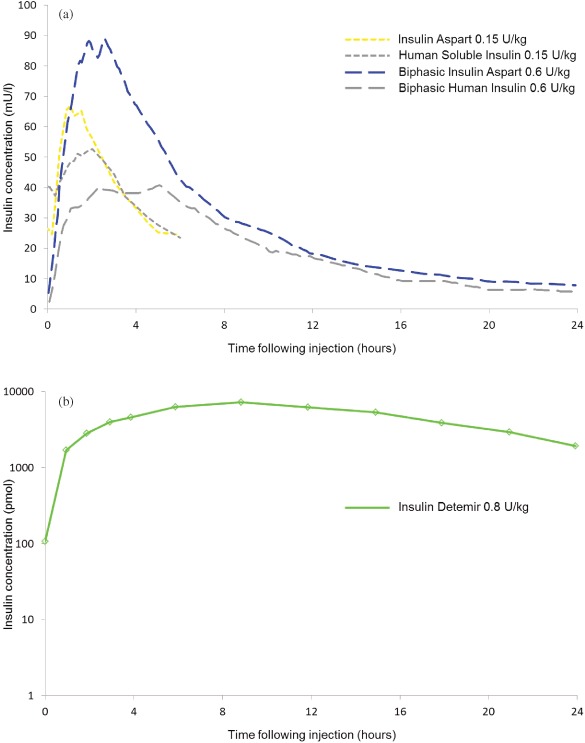
Insulin concentration from published pharmacokinetic studies conducted in patients with type 2 diabetes (a) insulin aspart (0.15 U/kg) [37], human soluble insulin (0.15 U/kg) [37], biphasic insulin aspart 30/70 (0.6 U/kg) [36], and biphasic human insulin 30 (0.6 U/kg) [36] and (b) insulin detemir (0.8 U/kg) [35].

Figure 2 shows the pharmacokinetic profiles of rapid-acting, biphasic and long-acting insulin analogues exclusively evaluated in patients with type 2 diabetes [35–37]. The profiles of human soluble and biphasic human insulin have been included for reference [37,38]. The rapid acting insulin analogue profiles mimic the normal physiological insulin secretion in response to meals better than regular human insulin, with a faster onset and higher maximal concentration [37]. The long-acting insulin analogue profiles, however, despite being an improvement in relation to NPH, are not entirely flat and do not remain consistent over a 24-h period. Both biphasic insulin analogues show more rapid absorption relative to biphasic human insulin and clearly distinguishable peak concentrations proportional to the dose of the soluble rapid-acting component [38]. However, the return to basal insulin levels is slow and uneven. This part of the biphasic insulin profile is commonly referred to as the ‘shoulder’, which occurs as a result of the interaction between soluble and protaminated insulin molecules [37,39].

One of the other interesting observations is that the absolute quantities of insulin measured vary, most notably between insulin detemir and the other insulin analogues. The higher insulin concentrations seen with insulin detemir reflects both the free and albumin bound (99% in plasma) insulin concentration, however, only the free insulin is available to interact with the target insulin receptor.

## Pharmacodynamics

In many ways, the shape of the desired pharmacodynamic insulin profile is similar to the pharmacokinetic profile, that is rapid onset and offset for the rapid-acting insulin analogues, and a long, flat and steady profile for long-acting insulin analogues.

One of the most commonly performed pharmacodynamic studies of insulin is the clamp study [40]. In a clamp study, subjects’ blood glucose levels are kept within a specific range or under a certain threshold measurement by infusing intravenous glucose. The amount of glucose infused to maintain blood glucose levels represents the amount of glucose uptake into the cells induced by insulin receptor activation. This is referred to as the GIR. Results of pharmacodynamic studies of patients with type 1 diabetes are easier to interpret as almost all glucose lowering is attributed to the study insulin. As already eluded to, clamp studies may employ different glycaemic thresholds and use different glucose-monitoring techniques. To minimize potential human error, the Biostator automates the process of monitoring and infusing glucose. This is important as over-infusion of glucose would result in a peak in the measured profile and also consume more insulin potentially resulting in an apparently shorter duration of action and vice versa. As with the array of differing definitions of hypoglycaemia, the lack of consensus on the way in which pharmacodynamic clamp studies are performed is a source of confusion. Through variation in glucose monitoring techniques, the choice of clamp glucose thresholds, the definition of end of action, the timing of injections in relation to the start of the clamp and the differing protocols with respect to the use of insulin infusion to switch off hepatic gluconeogenesis, published clamp studies are very difficult to compare relative to each other.

**Figure 3:**
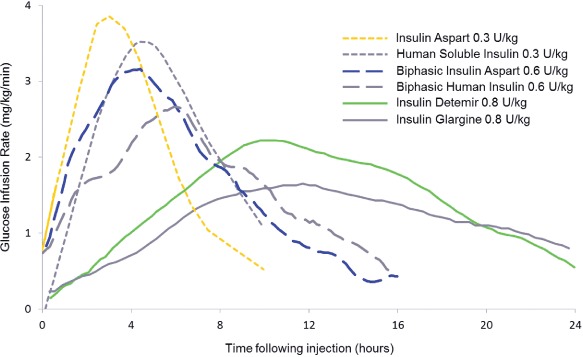
Glucose lowering effect from published pharmacodynamics studies conducted in patients with type 2 diabetes as measured by the glucose infusion rate (GIR) for insulin aspart (0.3 U/kg) [41], human soluble insulin (0.3 U/kg) [41], biphasic insulin aspart 30/70 (0.6 U/kg) [36], biphasic human insulin 30 (0.6 U/kg) [36], insulin detemir (0.8 U/kg) [35] and insulin glargine (0.8 U/kg) [35]. All insulin preparations were administered at time = 0 h.

Figure 3 presents GIR profiles of the insulin analogues obtained in patients with type 2 diabetes [35,36,41]. In patients with type 2 diabetes, both insulin lispro and insulin aspart administered immediately before meals have showed an 18–48% reduction in the rise of the 2-h postprandial glucose relative to human insulin given 30 min before the meal [37,42,43]. Insulin lispro and insulin glulisine have been shown to be equivalent on all glucose infusion measures in patients with type 2 diabetes, with the exception of a 0.5 mmol/l lower glucose excursion with insulin glulisine in type 2 patients with obesity [44]. There are no other studies comparing rapid-acting insulin analogues in patients with type 2 diabetes, or comparisons of the pharmacology of rapid-acting insulin analogues in type 2 relative to type 1 diabetes.

The pharmacodynamics of insulin lispro mix 25 has been derived from cross-over trials in patients given a standard test meal [45,46], which resulted in a lower peak rise in serum glucose compared with biphasic human insulin and NPH. In a similar experiment, biphasic insulin aspart 30 was found to have 44 and 34% lower postprandial glucose concentrations compared with biphasic human insulin 30 after breakfast and dinner, respectively [47]. These findings are consistent with the differences between rapid insulin analogues and human insulin. Furthermore, for those patients requiring a higher proportion of rapid-acting insulin, there is good evidence showing the relative increases in early postprandial glucose utilization with the higher ratio biphasic preparations (e.g. biphasic insulin aspart 50/50 and 70/30 [48–50], and biphasic insulin lispro 50/50 and 75/25). In addition, Parkner et al. [51] specifically compared normal and obese patients with type 2 diabetes with respect to insulin pharmacodynamics of biphasic insulin aspart and found no relationship between body mass index and blood glucose excursions. A single published clamp experiment [39] showed that for equivalent unit doses, the overall glucose lowering effect was greater for biphasic insulin aspart twice daily compared with insulin glargine once daily. These findings, however, are counterintuitive as an equivalent dose implies equivalent biological effect. Furthermore, a higher peak effect of the evening biphasic insulin aspart dose in this study led to recommendations by the authors to split doses 2/3 am and 1/3 pm to avoid increasing the risk of nocturnal hypoglycaemia.

As the function of long-acting insulin analogues is to provide basal coverage, ideally the GIR profiles should not only have no significant peak effect, but also show minimal day-to-day intra-patient variability. Whilst differences between patients are acceptable, variable responses to the same insulin dose within the same patient can increase the risk of hypoglycaemia and hyperglycaemia. Improved intra-patient variability has been shown with insulin detemir. In the study by Klein et al. [20] within-patient variability was significantly lower with insulin detemir than with insulin glargine in patients with type 2 diabetes, both in terms of the overall 24-h glucose lowering effect (47 vs. 215% variation) and the maximal glucose lowering effect (40 vs. 147% variation). Furthermore, although the duration of action increases with escalating insulin doses [20], currently available long-acting insulin analogues probably do not last 24 h, particularly at low doses. In single dose experiments, even doses of 0.8 U/kg achieved less than 24 h basal coverage in a significant proportion (up to 74%) of fasting patients with type 2 diabetes [35] and that almost twice this amount of insulin is required to achieve 24-h coverage under experimental conditions [20]. Lastly, several studies have reported clear peak effects 8–12 h after administration for both insulin glargine and insulin detemir in this patient population after a single dose administration [20,35,39].

Insulin preparations are most commonly studied in single dose, that is, a single dose is administered and the effects are measured. Single dose studies are more clinically relevant for assessing the pharmacology of rapid-acting insulin and are also useful in measuring onset of action. However, as peak effects may be exaggerated in single dose studies of long-acting insulin analogues and the concept of long-acting insulin is to provide continuous basal coverage, steady-state pharmacodynamic studies are probably more clinically relevant in this situation. Steady state is said to occur when there is an identical metabolic effect with subsequent doses. Essentially, the drug concentration is in equilibrium, where the amount metabolized to inactive metabolites and/or eliminated is the same as the amount of drug administered. Steady state can be achieved with all drugs and is dependent on the frequency of administration and the drug half-life. For drugs that follow first order kinetics (i.e. a constant fraction of the drug in the body is eliminated for each unit of time, which applies to the majority of drugs) drug concentrations reach to within 98% of the steady-state concentration at approximately three times the drug half-life. Unfortunately, to the authors’ knowledge, there are no published studies of steady-state insulin pharmacology in patients with type 2 diabetes.

In general, the magnitude of the peak in the glucose lowering effect of long-acting insulin is inversely proportional to the duration of action. For example, for the same dose, the peak effect of NPH is higher than those for the longer duration insulin glargine and insulin detemir. By slowing absorption further conditions for steady state are improved and thus the profile can be made flatter and more consistent with the basal insulin replacement/supplementation concept.

## Conclusions

Human insulin preparations including soluble, protaminated/precipitated and biphasic variants are appreciated to have limitations in terms of variability of absorption and thus glucose lowering action. Insulin analogues provide clinicians the opportunity to more closely emulate normal insulin physiology and to select different insulin regimens depending on patient preferences and lifestyle, thus making insulin treatment less restrictive to different circumstances. Faster onset of action of rapid acting insulin analogues has improved postprandial glycaemic control in patients with type 2 diabetes [37,38,47,52–54]; and more predictable glycaemic lowering profiles of the insulin analogues have also led to reductions in reported nocturnal hypoglycaemia, particularly comparing long-acting insulin analogues with NPH. A move away from suspensions of crystals containing protamine–insulin complexes and *in vivo* precipitation as the principal method of prolonging insulin absorption, has led to further reductions in within-patient variability [55,56].

Improvements can still be made, however, particularly in the area of long-acting insulin analogues, where effective once daily clinical use might not be possible in all patients and may require patients to administer insulin at a specific time of day which may not always be convenient or remembered. In addition, whilst clinicians have come to expect between subject variations because of different insulin requirements and hence the need for individual titration based on frequent blood glucose monitoring, a more consistent profile between individuals would be expected to simplify management. Even with the insulin analogues, successful treatment with any regimen still requires careful individual titration according to frequently measured blood glucose. This in itself is a problem as titration is a time consuming, continuous and often frustrating process; and patients are often punished in terms of deterioration in glycaemic control following even minor errors in dosing.

Finally, whilst biphasic insulin analogues have been shown to be associated with a lower incidence of nocturnal hypoglycaemia compared with human mix insulin and allow for intensification from once, to twice or thrice daily dosing, the protaminated/precipitated component does not provide the duration of action or profile for physiological basal insulin replacement. In essence, biphasic insulin is a mixture of rapid and intermediate acting insulin, that is, carrying the same limitations as the individual components. Unfortunately, neither insulin glargine nor insulin detemir are suitable for mixing with other insulin analogues as this mixing substantially alters their pharmacokinetic properties.

Further improvements in reducing the pharmacological variability of rapid- and long-acting insulin would be expected to lower the risk of hypoglycaemia and hyperglycaemia, as well as simplify and perhaps also encourage, optimal insulin titration in real-life clinical practice. Extending the duration of insulin effect would also allow for greater flexibility around the time of dosing and potentially reduce the frequency of blood glucose monitoring.
